# Two-Step Laparoscopic Surgery for a Patient with Synchronous Double Cancer of the Colon and Stomach Accompanied by Severe Chronic Obstructive Pulmonary Disease

**DOI:** 10.1155/2013/246515

**Published:** 2013-09-04

**Authors:** Kazuhito Yajima, Shin-Ichi Kosugi, Yosuke Kano, Takaaki Hanyu, Hiroshi Ichikawa, Takashi Ishikawa, Hitoshi Nogami, Toshifumi Wakai

**Affiliations:** Division of Digestive and General Surgery, Niigata University Graduate School of Medical and Dental Sciences, 1-757 Asahimachi-Dori, Niigata 951-8510, Japan

## Abstract

Laparoscopic treatment strategies for synchronous intra-abdominal malignancies have not yet been standardized. We report a successful case of two-step laparoscopic surgery for synchronous double cancer of the colon and stomach accompanied by severe chronic obstructive pulmonary disease (COPD). A 66-year-old man with COPD was diagnosed as having advanced colon cancer and early gastric cancer. On admission, he could not go upstairs (Grade III according to the Hugh-Jones classification) and his forced expiratory volume in 1 second was 600 mL (35.9%). The patient initially underwent laparoscopy-assisted sigmoidectomy with D3 lymphadenectomy, followed by laparoscopy-assisted distal gastrectomy with D1 lymphadenectomy 68 days later. The patient's each postoperative course was uneventful with no pulmonary complications, and the patient was discharged 9 and 11 days after the first and second operations, respectively. The present case demonstrates that two-step laparoscopic surgery may be a safe and feasible surgical procedure for high-risk patients with synchronous intra-abdominal malignancies.

## 1. Introduction

Laparoscopic surgery is recognized as a general surgical procedure for intra-abdominal malignancies [1, 2]. Recent publications have been reported on laparoscopy-assisted combined resection in patients with synchronous gastric and colorectal cancer [3–8]. However, laparoscopic treatment strategies for synchronous intra-abdominal malignancies have not yet been standardized, especially in patients with severe pulmonary comorbidity. Herein, we report on a two-step laparoscopic surgery in a patient with synchronous double cancer of the colon and stomach accompanied by severe chronic obstructive pulmonary disease (COPD).

## 2. Case Report

A 66-year-old man presented to the local hospital with a chief complaint of frequent loose stools and difficulty in defecation. He had smoked 40 cigarettes per day for 35 years, with his Brinkman index of 1400, and he has been having a history of COPD since 1999. Colonoscopy revealed a circumferential tumor in the sigmoid colon, with pathological examination indicating well-differentiated adenocarcinoma. The clinical diagnosis was advanced sigmoid colon cancer without nodal metastasis on enhanced abdominal-pelvic computed tomography. Screening the upper gastrointestinal endoscopy revealed a superficial depressed tumor in the greater curvature of the lower third of the stomach with pathological examination indicating well-differentiated adenocarcinoma. Submucosal invasion was clinically determined on endoscopy and upper gastrointestinal series; accordingly, surgical resection was recommended for both the sigmoid colon cancer and gastric cancer. On admission, the patient could not go upstairs (Grade III according to the Hugh-Jones classification) and breathed through pursed lips at rest. His vital capacity was 2530 mL (73.3%) and his forced expiratory volume in 1 second (FEV_1.0_) was 580 mL (35.9%). Chest radiography revealed increased lung transparency and cor pulmonale. These findings Compatible with COPD. Arterial blood gas analysis on admission revealed pH 7.397, PO_2_ = 79.3 mmHg, and PCO_2_ = 43.2 mmHg.

**Figure 1:**
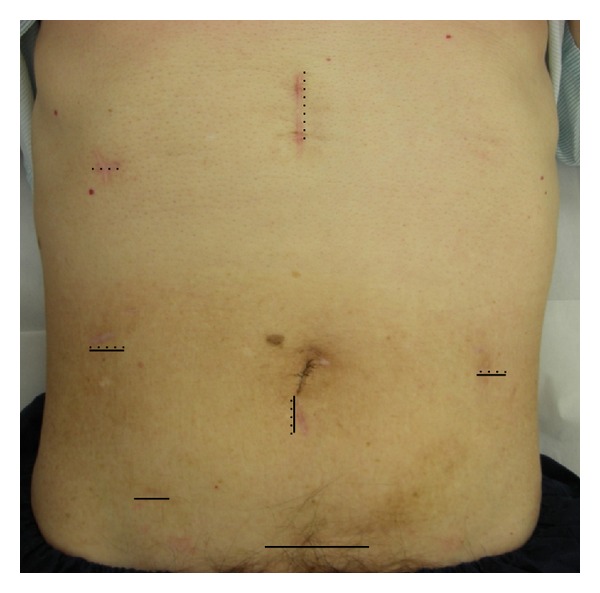
Port sites and minilaparotomy incision after sigmoidectomy and gastrectomy. Laparoscopic-assisted sigmoidectomy was performed via four ports and a 5 cm mini-laparotomy of the lower abdomen (solid lines). Laparoscopy-assisted distal gastrectomy was performed via four ports and a 4 cm mini-laparotomy of the upper abdomen (dotted lines).

Because of the diagnosis of advanced colon cancer and early gastric cancer, we decided to perform a two-step laparoscopic surgery to shorten the operation time and to avoid postoperative pulmonary complications. After admission, the patient underwent respiratory rehabilitation consisting of 3 weeks training using a TRIFLO II (Philips Respironics CK, Tokyo, Japan) and inhalation therapy. In September 2009, the patient underwent laparoscopy-assisted sigmoidectomy with D3 lymphadenectomy using four ports and the pneumoperitone method with an intra-abdominal pressure of 8 mmHg. Lymphadenectomy was performed using the medial-to-lateral approach [9] with resection margins of 10 cm on both the oral and anal sides of the tumor. The resected specimen was removed from a 5 cm incision in the lower abdomen (Figure 1). The colorectal anastomosis was laparoscopically performed by the double-stapling technique using circular stapler (DST Series EEA Stapler 28; Covidien Japan, Tokyo, Japan). The operation time was 177 min and the volume of blood lost was 5 mL.

**Figure 2:**
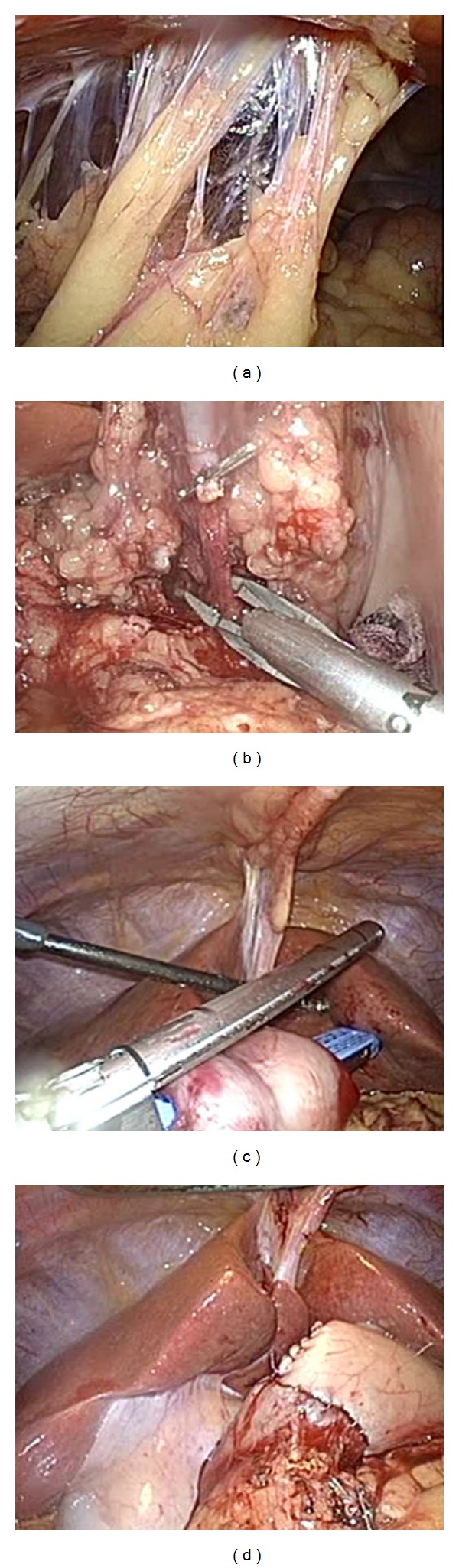
Intra-abdominal findings during laparoscopy-assisted distal gastrectomy with lymphadenectomy. (a) There was minimal adhesion of the omentum at the umbilical port site. (b) Dissection of lymph node stations 7 (lymph nodes along the trunk of left gastric artery). (c) Transection of the duodenum: the duodenum was cut nearly distal to the pylorus using an endoscopic liner stapler (GIA stapling system, blue; Covidien, Tokyo, Japan). (d) Billroth I reconstruction was performed by a hemi-double-stapling technique using circular stapler (DST Series EEA Stapler 28; Covidien Japan).

Sixty-eight days after the initial surgery for colon cancer, the patient underwent laparoscopy-assisted distal gastrectomy (LADG) using four ports and the pneumoperitoneal method with an intra-abdominal pressure of 8 mmHg. The first trocar was inserted into the abdominal cavity from the right lower quadrant by open method. There was minimal adhesion of the omentum at the umbilical port site (Figure 2(a)). D1 lymphadenectomy was performed by an usual method (Figure 2(b)), and transection of the duodenum and stomach was cut using an endoscopic liner stapler (GIA stapling system, blue; Covidien, Tokyo, Japan, Figure 2(c)). Billroth I reconstruction was performed by the hemi-double-stapling technique using circular stapler (DST Series EEA Stapler 28; Covidien Japan, Figure 2(d)) [10]. Removal of the resected stomach and anastomosis were performed through a 4 cm incision in the upper middle abdomen (Figure 1). The operation time was 136 min and the estimated blood loss was 35 mL. Intra-abdominal adhesion was minimal and, more importantly, gastrectomy could be performed safely and easily even though it was the second major surgery.

For both surgeries, the patient was under general anesthesia induced by propofol (1% Diprivan Injection; AstraZeneca, Osaka, Japan), vecuronium bromide (Musculax Intravenous; Schering-Plough, Osaka, Japan), and epidural anesthesia using ropivacaine hydrochloride hydrate (Anapeine Injection; AstraZeneca). The patient was extubated immediately in the operating theatre after recovering from anesthesia. The patient was managed postoperatively in the intensive care unit until postoperative day (POD) 1. The patient's each postoperative course was uneventful and he was discharged on POD 9 and 11 after the first and second operations, respectively. The patient is alive with no evidence of recurrence of either tumor 24 months after the initial colonic surgery and has a good quality of life without impairment of pulmonary dysfunction.

**Figure 3:**
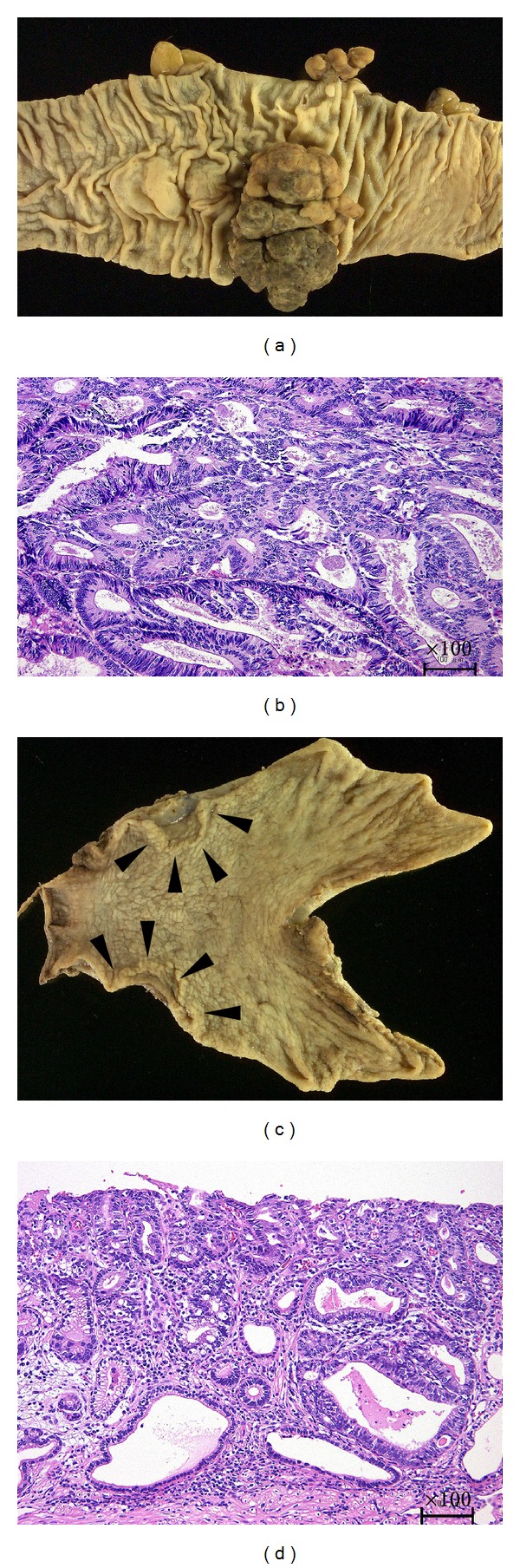
Macroscopic and microscopic findings of the sigmoid colon and gastric cancers. (a) The sigmoid colon contained a nearly circumferential polypoid tumor. (b) Histological examination revealed well-differentiated adenocarcinoma invading the subserosa (hematoxylin and eosin; original magnification, ×100). (c) The stomach contained a superficial depressed and elevated type tumor in the greater curvature of the lower third of the stomach (black arrowheads). (d) Histological examination revealed well- to moderately differentiated adenocarcinoma confined to the mucosa (hematoxylin and eosin; original magnification, ×100).

The resected sigmoid colon contained a nearly circumferential polypoid tumor, 58 × 38 mm in size (Figure 3(a)). Histological examination revealed well-differentiated adenocarcinoma invading the subserosa (Figure 3(b)), with no lymph node metastasis, which was pathologically classified as Stage II [11]. The resected stomach contained a superficial depressed and elevated type tumor, 25 × 20 mm in size (Figure 3(c), black arrow head). Histological examination revealed well-differentiated to moderately differentiated adenocarcinoma to the depth of the mucosa (Figure 3(d)), with no lymph node metastasis, which was pathologically classified as Stage IA [12].

## 3. Discussion

This is the first report of two-step laparoscopic resection for a patient synchronous double cancer of the colon and stomach accompanied by severe pulmonary comorbidity. Although the patient's respiratory function was very poor, the postoperative course after both operations was uneventful, with no pulmonary complications. The present case demonstrates that the two-step laparoscopic surgery may be a safe and feasible surgical technique for high-risk patients with synchronous intra-abdominal malignancies.

Laparoscopic gastrectomy is the accepted procedure for the treatment of early gastric cancer because of its superiority to conventional open gastrectomy in terms of less postoperative pain, less blood loss, and earlier postoperative recovery [13]. Currently, across all the centers registered in Japan to perform endoscopic surgery, 27.9% of operations for gastric cancer are performed laparoscopically [14]. For colon cancer, laparoscopic surgery is even more common, with a nationwide survey in Japan reporting that 46.8% of colorectal surgeries are performed laparoscopically [14]. In our division, we have performed laparoscopic gastrectomy in patients with clinical T1 gastric cancer with no lymph node metastasis since July 2002, and laparoscopic colectomy has been performed in patients with colorectal cancer with no clinical evidence of invasion to adjacent organs since August 1995.

The treatment for patients with synchronous colon and gastric cancer has been one-step resection. Although the precise surgical procedure depends on the location of both the gastric and colorectal cancers, the biggest disadvantage associated with conventional one-step open surgery is the extensive surgical manipulation. To reduce the invasiveness of the resection, laparoscopic simultaneous resections were recently introduced for patients with these double cancers. To characterize laparoscopic simultaneous resections and to determine any problems associated with the procedure, we searched the PubMed database using the keywords “laparoscopy,” “gastric cancer,” and “colorectal cancer.” As of December 2012, there were six reports on 16 patients, including reference lists, describing laparoscopic surgery for synchronous gastric and colorectal cancer [3–8]. All 16 cases and our case are summarized in Table 1.

As indicated in Table 1, LADG was selected for 11 patients with gastric cancer, laparoscopy-assisted total gastrectomy was performed in two patients, laparoscopy-assisted pylorus-preserving gastrectomy was performed in another two patients, and laparoscopy-assisted proximal gastrectomy was performed in one patient. Various procedures were performed for colonic resection, which included right colectomy in six patients, sigmoidectomy in four patients, low anterior resection in another four patients, and partial resection, left colectomy, and ileocecal resection in one patient each. The median operation time was 386 min (range, 263–746 min), and median blood loss was 147.5 mL (range, 15–500 mL). The median postoperative hospital stay was 14 days (range, 6–51 days). There were four postoperative complications including wound infection, surgical site infection, enteritis, and gastric fullness in one patient each. On the basis of these outcomes, it appears that the less-invasive laparoscopic surgery is useful in the case of both colon and gastric cancers and is associated with good short-term outcome.

Although the laparoscopic approach is a feasible procedure for synchronous gastric and colorectal cancer, there are two major limitations. First, the simultaneous laparoscopic procedure is time consuming: it took longer than 5 h to be completed in 14 (87.5%) of the 16 patients, with >12 h (746 min) required in one patient (Table 1). Second, there seem to be difficulties in determining port sites and small skin incisions for the resection and anastomosis. Tokunaga et al. [6] reported that the minilaparotomy and port sites depend on the location of both the gastric and colonic tumors, with seven patients undergoing simultaneous laparoscopic surgery in their report requiring three minilaparotomy wounds. Matsui et al. [5] reported that the combined resection of the right-side colon and stomach was relatively easy and was performed sharing the same minilaparotomy and ports. In the present case, if the operation had been performed simultaneously, totally laparoscopic distal gastrectomy and sigmoidectomy with minilaparotomy extending umbilical port site may have been needed because the colon cancer was located in the sigmoid colon.

Laparoscopic simultaneous resection of gastric and colorectal cancer in patients with comorbidities, especially severe pulmonary complications, has not been reported. Carbon dioxide (CO_2_) is generally used in laparoscopic surgery to create a pneumoperitoneum because it is quickly absorbed from the peritoneal cavity into the circulation; however, there is little known about its effect on patients with severe COPD. In general, pulmonary function is impaired following major abdominal surgery because of limited functioning of the diaphragm. In a retrospective study, Chang et al. [15] reported on 61 patients with COPD undergoing laparoscopic gastrectomy. They found significant changes in end-tidal CO_2_ and P_a_CO_2_ during laparoscopic gastrectomy, but the procedure could be performed safely in patients with mild to moderate COPD. Because our patient had very severe COPD according to the Chang's criteria [15], with FEV 1.0 ratio of 23%, we intended to perform the two-step laparoscopic surgery for synchronous double cancer of colon and stomach. We were able to safely complete laparoscopy-assisted colectomy at first the operation, followed by LADG at the second operation with no postoperative pulmonary complications.

## 4. Conclusions

In conclusion, herein we report on successful two-step laparoscopy resection for a patient with synchronous double cancer of the colon and stomach accompanied by severe pulmonary comorbidity. Two-step laparoscopic surgery is useful because it reduces the operative time and minimizes the length of the surgical incision. This strategy may be safe and feasible for high-risk patients with synchronous intra-abdominal malignancies.

## 5. Conflict of Interests

The authors declare there is no conflict of interests.

## Figures and Tables

**Table 1:** Reported cases of laparoscopic resection for synchronous double cancer of the colon and stomach.

Case	Author	Age (y)	Sex	one- or two-step	Laparoscopic procedures (colectomy/gastrectomy)	Operation time (min)	Blood loss (mL)	Hospital stay (days)	Complications
1	Tessier and Harold [3]	72	M	one-step	RC/DG	378	200	6	None
2	Zhu et al. [4]	55	M	one-step	LAR/DG	270	120	13	None
3	Matsui et al. [5]	72	F	one-step	Partial/PPG	474	145	14	None
4	Matsui et al. [5]	67	M	one-step	RC/DG	432	400	15	None
5	Matsui et al. [5]	71	M	one-step	LAR/PG	746	150	ND	Wound infection
6	Tokunaga et al. [6]	66	M	one-step	LAR/DG	365	100	15	SSI
7	Tokunaga et al. [6]	71	M	one-step	S+RC/DG	363	65	12	None
8	Tokunaga et al. [6]	67	M	one-step	RC/DG	350	15	11	None
9	Tokunaga et al. [6]	77	M	one-step	S/TG	439	160	13	None
10	Tokunaga et al. [6]	72	M	one-step	RC/TG	576	250	19	Enteritis
11	Tokunaga et al. [6]	72	M	one-step	LC/DG	386	15	51	Gastric fullness
12	Tokunaga et al. [6]	66	M	one-step	LAR/PPG	263	24	16	None
13	Nishikawa et al. [7]	84	F	one-step	S/DG	315	80	15	None
14	Nishikawa et al. [7]	70	M	one-step	ICR/DG	340	300	13	None
15	Nishikawa et al. [7]	58	M	one-step	S/DG	495	440	10	None
16	Lee et al. [8]	78	M	one-step	RC/DG	400	500	17	None
17	Our case	66	M	two-step	S/DG	177/136	5/35	9/11	None/none

One-step or two step: one- or two-step surgery; DG: distal gastrectomy; PPG: pylorus-preserving gastrectomy; PG: proximal gastrectomy; TG: total gastrectomy; RC: right colectomy; LAR: low anterior resection; S: sigmoidectomy; LC: left colectomy; ICR: ileocecal resection; SSI: surgical site infection.
